# Role of Scleroglucan Produced by *Sclerotium rolfsii* in Shaping the Microstructure, Rheology, and Flavour Profile of Full-Fat Yoghurts

**DOI:** 10.3390/molecules30244696

**Published:** 2025-12-08

**Authors:** Marika Magdalena Bielecka, Aneta Zofia Dąbrowska, Małgorzata Anna Majcher, Marek Aljewicz

**Affiliations:** 1 Department of Dairy Science and Quality Management, Faculty of Food Science, University of Warmia and Mazury in Olsztyn, Oczapowskiego 7, 10-719 Olsztyn, Polandanetazj@uwm.edu.pl (A.Z.D.); 2 Faculty of Food Science and Nutrition, Poznań University of Life Sciences, Wojska Polskiego 31, 60-624 Poznań, Poland

**Keywords:** volatile compounds, protein–polysaccharide interactions, starter culture metabolism, organic acid, β-glucan

## Abstract

This study evaluated the effects of different concentrations (0.25%, 0.5%, and 1.0% *w*/*w*) of highly purified (90%) β-glucan (scleroglucan—SCGL) produced by *Sclerotium rolfsii* on the physicochemical, rheological, microbiological, and sensory properties of full-fat yoghurt (3.2% fat). The fermentation dynamics, titratable acidity, apparent viscosity, hardness, adhesiveness, colour, microstructure, and volatile compound profiles of the studied yoghurts were analysed. The addition of SCGL increased gel hardness and viscosity, while preserving its pseudoplastic flow behaviour (*n* = 0.10–0.15). In samples containing 1.0% SCGL, yield stress (τ_0_) increased from 0 Pa in the control to 739 Pa after 28 days of storage, pointing to the formation of a dense protein–polysaccharide network. The analysed polysaccharide slowed down lactose hydrolysis and acidification, but increased the counts of *Streptococcus thermophilus* (7.7 log CFU·g^−1^) compared to the control (5.8 log CFU·g^−1^). The volatile compound analysis showed increased acetaldehyde (5.6 mg·L^−1^) and diacetyl (5.0 mg·L^−1^) levels and reduced acetoin (~1.0 mg·L^−1^) concentration, which enhanced the intensity of the buttery aroma. The sensory evaluation revealed that yoghurts containing 1% SCGL had the most desirable smooth consistency and a balanced, fresh aroma, whereas yoghurts with lower SCGL concentrations (0.25–0.5%) were characterised by a mealy mouthfeel and thinner consistency. Scleroglucan proved to be an effective natural stabiliser and flavour modulator that improved the structure, stability, and sensory quality of full-fat yoghurts.

## 1. Introduction

Yoghurt, one of the most widely consumed fermented dairy products worldwide, exhibits substantial regional variability in intake. In 2022, global per capita consumption was estimated at approximately 11.9 kg per year [1], while in several European and Middle Eastern countries consumption was considerably higher—for example, around 37 kg in Israel and 25–33 kg in Switzerland, Finland, and Bulgaria. Patterns of consumption on this scale indicate that yoghurt is firmly established in daily diets, creating continued interest in improving its texture, stability and sensory characteristics. However, consumer expectations have increasingly shifted toward clean-label, minimally processed formulations, which has intensified interest in natural structuring agents capable of improving both texture and health value [2].

Yoghurt is a complex colloidal system whose physicochemical and sensory properties arise from compositional and technological factors. During fermentation and storage, the progressive rearrangement of the casein–fat network—driven by isoelectric coagulation associated with lactic acid accumulation—plays a key role in shaping texture, microstructure, water-holding capacity, and overall consumer perception [3].

In addition to its technological relevance, yoghurt consumption has been associated with several health benefits. These include improved lactose digestion in lactose-intolerant individuals, modulation of the gut microbiota, enhanced immune responses, and support for metabolic and gastrointestinal health. Such effects are related to the presence of viable lactic acid bacteria (LAB), bioactive peptides released during fermentation, and the improved bioavailability of nutrients such as calcium and B vitamins. Regular intake has also been linked to reduced risk of type 2 diabetes, im-proved weight management and enhanced cardiometabolic profiles [4].

In industrial yoghurt production, hydrocolloids and functional biopolymers are widely applied to improve water-holding capacity, reduce syneresis, and refine mouthfeel, thereby enhancing the structural integrity and sensory appeal of the final product [5,6]. Although stabilisers such as guar gum, locust bean gum, and carrageenan are effective, the growing preference for natural, clean-label, and multifunctional ingredients has increased interest in novel biopolymers that combine technological functionality with potential health benefits [7,8]. Microbial polysaccharides—including curdlan, xanthan, gellan, and scleroglucan (SCGL)—are particularly promising alternatives to plant-derived stabilisers.

Scleroglucan (SCGL), a β-(1→3)(1→6)-D-glucan produced by *Sclerotium rolfsii*, is characterised by high molecular weight (≈2–3 × 10^6^ Da) and a triple-helical conformation that confers excellent solubility, pseudoplastic flow behaviour, and remarkable thermal and pH stability [7,9]. Its molecular structure, stabilised by intramolecular hydrogen bonding, enables effective thickening and stabilisation at low concentrations, while offering prebiotic potential comparable to other β-glucans [10,11]. Compared with plant- or marine-derived polysaccharides, microbial exopolysaccharides such as SCGL exhibit advantages including sustainability, high production yields (0.0022–100 g·L^−1^), and greater process reproducibility [12]. Importantly, SCGL’s triple-helical conformation sets it apart from many other microbial polysaccharides and is likely to influence how it associates with milk fat and casein micelles.

β-Glucans also interact with milk proteins and lipids, contributing to the formation of structurally complex dairy matrices [13,14,15]. These interactions may alter fermentation kinetics, acidification behaviour, and the formation or retention of key volatile compounds such as acetaldehyde, diacetyl, and acetoin. Importantly, the presence of milk fat influences metabolic pathways responsible for volatile compound synthesis and affects the development of texture and rheological properties, which may determine how yoghurt responds to the addition of polysaccharides. Despite these considerations, the capacity of SCGL to modulate fermentation behaviour, acidification pathways, textural development, and flavour formation in yoghurt—particularly in full-fat formulations—remains insufficiently explored.

Previous research had demonstrated that dietary fibres and β-glucans from cereal or microbial sources can influence yoghurt structure, viscosity, and fermentation kinetics, although their effects depend strongly on molecular weight, concentration, and interactions with milk proteins [14,16]. Recent findings by Aljewicz et al. (2025) had shown that SCGL acted as an effective structure-forming agent in low-fat yoghurt, having slowed acidification, increased viscosity and gel firmness, eliminated syneresis, modified volatile profiles and maintained starter culture viability, with 1% SCGL achieving the highest sensory acceptability [13]. However, extrapolation of these results to full-fat yoghurt is not straightforward, because higher fat content alters casein–fat–polysaccharide interactions, changes metabolic fluxes for volatile compound synthesis, and may lead to different rheological responses. Analogous effects in full-fat yoghurt—where higher fat content may profoundly modify metabolic pathways and structural development—have not yet been characterised.

In light of this, the present study examined the effects of SCGL on fermentation, physicochemical characteristics and flavour development in full-fat yoghurt. We hypothesised that the incorporation of SCGL, a microbial β-glucan with thickening and stabilising properties, would modify fermentation kinetics and the formation of the yoghurt matrix, leading to measurable differences in acidification patterns, organic acid composition, rheological behaviour, and microbial dynamics compared with control samples. Therefore, the aim of this study was to elucidate the influence of microbial SCGL on fermentation behaviour, volatile profiles, and sensory quality in full-fat yoghurt, and to advance understanding of the biochemical mechanisms by which β-glucans contribute to flavour formation and structural development in fermented dairy matrices.

## 2. Results and Discussion

### 2.1. Chemical Composition of Yoghurts

The chemical composition of the analysed yoghurts was typical of full-fat fermented products. The fat content and total dry matter content were standardised to the values characteristic of these types of products. Despite standardisation, slight differences in protein content (3.57–4.23%) and dry matter content (13.95–14.23%) were observed between the samples (Table 1). Scleroglucan was used in the experimental yoghurts at concentrations of 0.25%, 0.5%, and 1%, while skimmed milk powder (SMP) was used in the control samples to increase their dry matter content. Dry matter content was highest in yoghurts with the lowest concentrations of SMP and SCGL (0.25%), while lowest in products with the highest concentrations of these additives (1%) (Table 1). This inverse relationship may indicate that higher concentrations of the applied additives affected the water-binding capacity or the hydration behaviour of the protein–polysaccharide matrix, causing a slight dilution effect.

Protein content was generally higher in the control yoghurts than in those containing SCGL, which could be attributed to the direct contribution of SMP protein, potential dilution effects, or interactions in the protein–polysaccharide matrix of the experimental samples. The fat content of all samples was standardised to 3.2%. The results indicate that both the type of additive (SMP vs. SCGL) and its concentration can affect the overall chemical composition of yoghurt, thus potentially influencing its textural properties and sensory characteristics.

These observations suggest that although lower concentrations of SCGL increase the dry matter content of yoghurt, higher concentrations may reduce dry matter content by changing the water-binding properties in the protein–polysaccharide system.

### 2.2. Effect of Scleroglucan on Yoghurt Colour

The analysis of colour parameters in the CIE-LAB space (Table 2) showed that the addition of SCGL significantly affected the colour of yoghurts, particularly in the initial period of storage. After three days of storage, yoghurts containing SCGL exhibited significantly lower lightness (L) values compared to the control samples (*p* < 0.05), indicating a darker shade. The greatest decrease in lightness was observed in the variant with 0.25% SCGL, where the L* value decreased from 87.81 (control) to 76.67 (*p* < 0.05). This change corresponds to a total colour difference in ΔE ≈ 11.2 (ΔL = −11.1; Δa = −0.8; Δb = −0.4), which is visually perceptible and confirms the significant effect of the additive on colour perception. The darkening effect was much less pronounced in yoghurts with higher SCGL concentrations (0.5% and 1%). In these variants, L* values were significantly lower than in the control only in the early stage of storage (*p* < 0.05), while differences between subsequent storage days were not statistically significant. After three days of storage, the total colour difference (ΔE) in the 1% SCGL variant was determined at only around 1.6, indicating a value close to the threshold of perceptibility to the human eye (ΔE < 2). This suggests that higher polysaccharide concentrations limited the initial darkening effect, probably by stabilising the gel structure and promoting a more uniform distribution of fat and protein phases. The colour differences between the control and experimental yoghurts gradually decreased on successive days of storage (up to 28 days). After 28 days, ΔE ranged from 0.3 to 1.5 in all samples, which is below the visual perception threshold, and the observed differences were not statistically significant (*p* > 0.05). During this period, only minor, non-significant differences were found in colour parameters L*, a*, and b*, regardless of additive concentration. The above can be explained by the stabilising effect of the fat phase, which increases the product’s brightness (L*) and masks subtle shade differences by scattering light in a colloidal system. These findings are consistent with the results of other studies investigating xanthan gum, guar gum, inulin, and β-glucans, which have shown that these additives exert indirect effects on the colour of dairy products, mainly by improving their texture, reducing syneresis, and promoting gel stabilisation [17,18,19,20].

### 2.3. Effect of Scleroglucan on Yoghurt Texture

The addition of SCGL significantly modified the textural properties of full-fat yoghurts, thus confirming that this additive is an effective natural stabiliser and structure-forming agent. Compared to the control samples, the hardness of the experimental yoghurts increased already in the early stage of storage, and this effect was intensified with an increase in additive concentration. After 28 days of storage, the hardness of 1% SCGL samples was maintained at 6.84 N, indicating the stability of the protein–polysaccharide network and no gel degradation during storage (Table 2). The hardness of the variants with lower SCGL concentrations (0.25–0.5%) also increased over time, confirming that even a small amount of SCGL strengthens the colloidal system, although this effect is dose-dependent. This mechanism probably can be linked to non-covalent hydrogen and hydrophobic interactions, which stabilise the connections between casein micelles and limit water mobility in gel pores [21]. At the same time, adhesiveness, measured as the negative value of the adhesion force, decreased in the experimental samples, especially those with SCGL concentrations above 0.5%. In the 1.0% SCGL variant, adhesiveness reached −19.22 N·s after 28 days of storage, pointing to the formation of a compact, homogeneous matrix with greater cohesion and lower surface viscosity (Table 2). These changes are characteristic of systems exhibiting effective water binding and spatial restructuring of the milk protein network, including yoghurts with the addition of with anionic natural gums [21]. When the results obtained for SCGL were compared with those for other polysaccharides, a similar trend was observed, but the dynamics of the system’s response differed. Pullulan also strengthened the gel and reduced syneresis at a concentration of 2%, but at 1%, it weakened the gel structure and increased whey separation [22]. Gellan gum formed denser protein clusters, improved water retention, and increased gel elasticity [21], whereas tara gum exhibited the strongest cross-linking effect at concentrations of 0.25–0.50 g L^−1^, above which excessive gelation or partial phase separation occurred [23]. In turn, barley β-glucans contributed to excessive hydration and gel weakening [24]. The textural response of full-fat yoghurts to SCGL addition is consistent with the behaviour of this polysaccharide in low-fat set-type systems, where 0.5–1.0% SCGL likewise eliminated syneresis and markedly increased firmness and cohesiveness throughout refrigerated storage, despite the reduced fat content [13]. In the above study, the relative increase in hardness and firmness was even more pronounced than in the present full-fat yoghurt, which supports the view that SCGL can partly compensate for the loss of fat-induced structure and creaminess in low-fat formulations while maintaining gel integrity. Taken together, the two datasets indicate that SCGL acts primarily as a structure-forming agent that reinforces the casein network irrespective of fat level, whereas milk fat modulates mainly the perception of smoothness and richness rather than the existence of a continuous gel.

### 2.4. Effect of Scleroglucan on Yoghurt Rheology

An analysis of the flow curves of yoghurts confirmed that the tested systems were best described by the Herschel–Bulkley model (R^2^HB = 0.853–0.989). In the control samples, the shear threshold was negligible (τ_0_ ≈ 0 Pa) after gentle stirring. In the SCGL variants, τ_0_ increased significantly after three days of storage (from approx. 221 Pa in 0.25% SCGL (S3) to approx. 480 Pa in 1% SCGL (S3)) and it increased by another 50–60% after 28 days of storage, which was associated with the formation and stabilisation of a shear-resistant protein–polysaccharide network. In 1% SCGL samples, a very high yield point was observed with a moderate increase in viscosity, suggesting that the system remained stable at rest but was more susceptible to liquefaction under shear stress (Table 3). This behaviour reflected a progressive densification of the protein network, in which the introduction of SCGL reduced the mobility of casein micelles and facilitated their rearrangement into a more continuous, shear-resistant structure. Scleroglucan chains form a triple helix that strengthens the casein network by forming hydrogen bonds and hydrophobic interactions with milk proteins. In whole milk, SCGL additionally stabilises the fat–water interface, promoting the formation of stable, evenly distributed clusters in the yoghurt gel. This explains why at 0.25–0.5% SCGL, τ_0_ increased with an improvement in the macrostructure, whereas at 1% SCGL, a very high yield point was observed without a proportional increase in viscosity. The structure remained stable at rest but liquefied easily under shear stress. Similar observations were made by Ge et al. (2022) who found that polysaccharides stabilise the gel structure by interacting with milk proteins [21].

The consistency index (k) and its variations over time reflected the interplay between the strengthening of the continuous phase and the stiffening of the fat–water interface. In the control yoghurts, k decreased by approximately 15–20% during storage, which was associated with the remodelling of the casein network and redistribution of water to the serum phase. Initially, this parameter was lower in 0.25–0.5% SCGL samples than in the controls (up to approx. 60%), but after 28 days of storage, k increased by 100–120% relative to the initial values, suggesting a slow “sealing” of the matrix by SCGL helical segments and stabilisation of the fat emulsion. This initial decrease in k reflected the fact that low SCGL concentrations interfered with early-stage micellar aggregation, leading to a more open network with lower continuous-phase viscosity, despite a simultaneous increase in yield stress resulting from improved network connectedness. After 28 days, k was lower in 1% SCGL samples than in the controls despite an increase in τ_0_ and hardness, indicating that gel stiffening was caused mainly by the strengthening of interphase connections rather than an increase in the viscosity of the continuous phase. A non-linear dose response was also reported in other polysaccharides: anionic gellan gum strengthened the elastic moduli through electrostatic bridging even at very low concentrations, whereas fillers such as inulin showed an effect only above a few percent [21].

The flow index (*n*) remained low (0.10–0.15), confirming the pseudoplastic nature of the tested systems—high resistance of the structure at rest and easy liquefaction under shear stress. The *n* values decreased during storage, pointing to gradual cross-linking and an increase in gel elasticity. Similar relationships were observed in yoghurts containing bacterial exopolysaccharides and in systems with xanthan gum, where increased cross-linking resulted in strong pseudoplasticity and increased gel stability [18].

Apparent viscosity (η) at shear rates of 50–100 s^−1^ was greatly affected by the concentration of SCGL and storage time. The values of η(50 s^−1^) and η(100 s^−1^) were 35–70% higher in 0.25–0.5% SCGL samples than in the controls, including after 28 days of storage, which contributed to a fuller perception of creaminess and reflected the progressive sealing of the protein matrix by SCGL. Despite the fact that 1% SCGL samples were characterised by the highest τ_0_, their η(50–100 s^−1^) values were more than 60% lower than in the controls, which points to high stability at rest and easy liquefaction under shear stress. This behaviour is characteristic of neutral β-glucans, which stabilise emulsions through steric interactions at the fat–water interface rather than by increasing the viscosity of the aqueous phase. It is conceivable that in full-fat yoghurt SCGL may adsorb onto fat droplets and form a steric protective layer, as shown in model oil-in-water emulsions stabilised with SCGL [25], which could con-tribute to decoupling yield stress from shear viscosity. Such steric effects would limit droplet coalescence and support the formation of a stable dispersed phase, potentially reinforcing the three-dimensional network at rest without proportionally increasing shear viscosity. The results of the rheological analysis were consistent with texture measurements. The variants with 0.25–0.5% SCGL, characterised by higher τ_0_ and moderately higher η(50–100 s^−1^) values, exhibited greater hardness and gel cohesion. After 28 days of storage, the hardness of all SCGL samples increased, most notably in the 1% SCGL variant, which confirms further cross-linking and structural stabilisation. Scleroglucan exhibits a hybrid mode of action by thickening the protein matrix, stabilising the fat–water interface, and improving phase integration. Unlike anionic polysaccharides (such as κ-carrageenan), which stiffen the gel through electrostatic interactions even at trace concentrations, neutral SCGL strengthens the structure through hydrogen bonding and steric effects. Therefore, compact and elastic gels were obtained at SCGL concentrations of 0.25–0.5%, whereas stronger stiffening was observed at 1% due to intense stabilisation of the fat–water interface, without com-promising gel cohesion.

### 2.5. Effect of Scleroglucan on Yoghurt Microbiology

**Figure 1:**
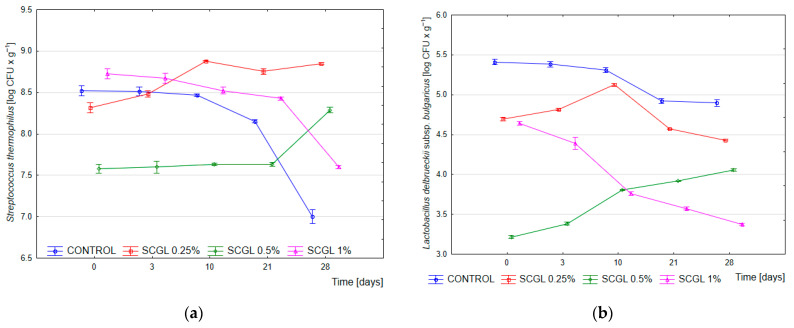
Average viability of starter cultures in yoghurt samples, measured immediately after fermentation (0 h) and during 28 days of refrigerated storage. (**a**) *Streptococcus thermophilus*; (**b**) *Lactobacillus delbrueckii* subsp. *bulgaricus*. Control—control samples; 0.25%, 0.5%, and 1% SCGL—yoghurt samples containing scleroglucan at the respective concentrations. Data are presented as mean values (*n* = 3), and error bars represent standard deviation.

After the completion of fermentation (time 0; pH 4.6), the count of *Streptococcus thermophilus* in the control samples averaged 8.52 log CFU·g^−1^, whereas in the SCGL variants, it ranged from 7.58 to 8.73 log CFU·g^−1^. After 28 days of storage, a marked decline was observed in the control, while the SCGL-containing samples maintained stable values of approximately 8.85 log CFU·g^−1^ (0.25%), 8.29 log CFU·g^−1^ (0.5%) and 7.60 log CFU·g^−1^ (1.0%). Two-way ANOVA confirmed a significant effect of SCGL concentration (*p* < 0.001, η^2^p = 0.945), storage time (*p* < 0.001, η^2^p = 0.800) and their interaction (*p* < 0.001, η^2^p = 0.957), indicating that the dynamics of *S. thermophilus* were strongly dependent on the level of the polysaccharide. The counts of *Lactobacillus delbrueckii* subsp. *bulgaricus* were lower than those of *S. thermophilus* across all treatments and declined systematically during storage, which was confirmed by highly significant effects of SCGL concentration (*p* < 0.001, η^2^p = 0.994), time (*p* < 0.001, η^2^p = 0.890) and their interaction (*p* < 0.001, η^2^p = 0.976) (Figure 1).

The obtained results were consistent with earlier reports on β-glucans, in which higher concentrations of polysaccharides reduced the growth of yoghurt lactobacilli and modified the course of acidification [14,26]. In the present study, SCGL in the full-fat milk matrix did not decrease the viability of *S. thermophilus*; on the contrary, it promoted its maintenance in the late storage period. This effect corresponded to observations from low-fat yoghurts enriched with SCGL, where no negative influence of the polysaccharide on starter culture viability was reported [13].

Compared with the previously examined low-fat system [13], the full-fat formulation used here introduced an additional factor—the milk fat globule and its membrane components (MFGM). According to the literature, MFGM phospholipids, glycolipids and membrane proteins alleviate acid-related and osmotic stress and enhance the resilience of lactic acid bacteria [27]. Therefore, the higher stability of *S. thermophilus* observed after 28 days of storage in this study may have resulted from the combined action of SCGL and the protective effect of the fat matrix, which distinguished this system from the previously examined low-fat yoghurt.

The lower counts of *L. delbrueckii* subsp. *bulgaricus* in samples with higher SCGL concentrations aligned with earlier descriptions of reduced activity of yoghurt lactobacilli in the presence of high-molecular-weight polysaccharides. The slowed acidification and reduced final acidity observed in the present experiment corresponded to previous findings for β-glucans, in which increased viscosity limited the diffusion of substrates and metabolites [14,26]. The denser protein–polysaccharide network most likely stabilised S. thermophilus by buffering local pH fluctuations, while being less favourable for *L. delbrueckii* subsp. *bulgaricus*, which is more sensitive to low pH and constrained metabolite diffusion.

### 2.6. Effect of Scleroglucan on Milk Acidification and Organic Acid Production in Yoghurt

**Figure 2:**
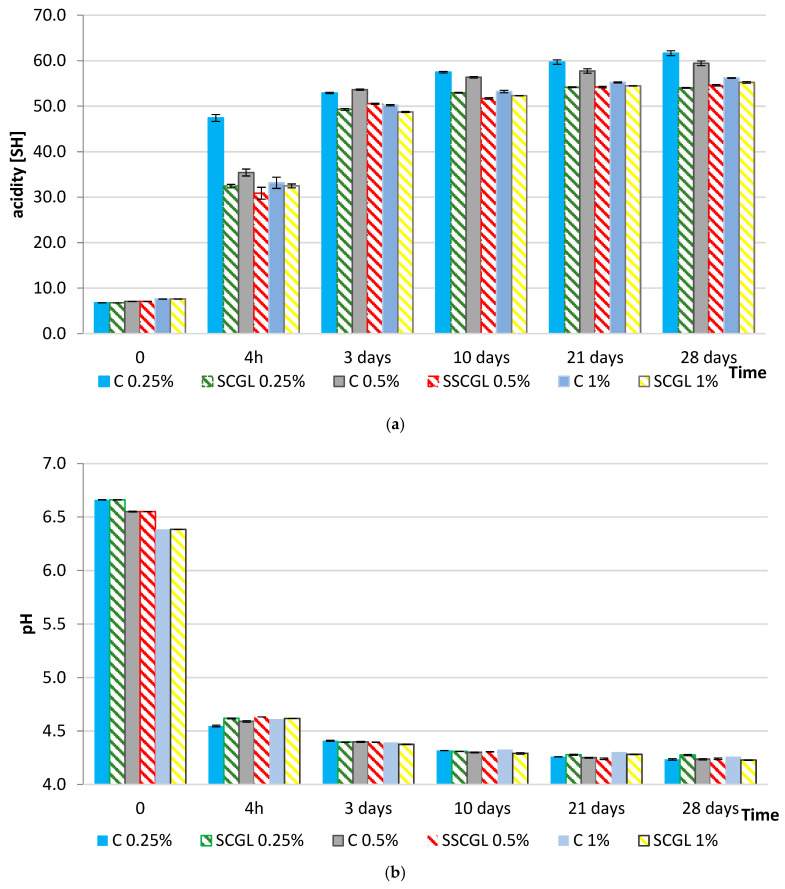
Changes in (**a**) pH and (**b**) titratable acidity (°SH) during fermentation and 28 days of refrigerated storage of full-fat yoghurts containing skimmed milk powder (SMP; 0%, 0.25%, and 1%; control samples, C) and scleroglucan (SCGL; 0.25%, 0.5%, and 1%; experimental samples). Values are presented as means ± SD (*n* = 3); differences are significant at *p* < 0.05.

During yoghurt fermentation, lactose had been rapidly metabolised by LAB, with the most intense hydrolysis occurring within the first 3 days (Figure 2). In the control samples, this process had coincided with a sharp decline in pH (from ~6.6–6.7 to 4.55–4.69 after 4 h) and a corresponding increase in titratable acidity (~50 °SH), reflecting efficient conversion of lactose into lactic acid. In contrast, both lactose utilisation and lactic acid accumulation had proceeded more slowly in SCGL-enriched yoghurts. This reduction in metabolic rate had aligned with the behaviour of the starter cultures observed earlier: *Streptococcus thermophilus* had remained metabolically active during storage in the presence of SCGL maintaining counts above 8 log CFU g^−1^ after 28 days in the 1% variant—whereas the control sample had exhibited a marked decline. Conversely, *Lactobacillus delbrueckii* ssp. *bulgaricus* had shown the greatest viability loss at the highest SCGL dose, likely due to the compact protein–polysaccharide matrix that had restricted nutrient diffusion and metabolite transport.

The pH profiles observed in the present study follow the characteristic three-stage acidification pattern described for yoghurt systems: a lag phase with slow pH decline, an exponential phase driven by rapid lactose conversion to lactic acid, and a final slowdown as the pH approaches its equilibrium value [28]. The moderated acidification in SCGL samples had reflected a lengthened lag phase and a reduced acidification rate during the exponential stage, which had been consistent with the slower progression of lactose hydrolysis and lactic acid accumulation observed in these formulations.

The slower acidification observed in SCGL-containing samples can additionally be attributed to diffusion limitations caused by the increased viscosity and density of the protein–polysaccharide network. The triple-helix conformation of scleroglucan had created a more crowded microenvironment that had reduced the mobility of lactose, amino acids and dissolved oxygen, thereby slowing the glycolytic activity of starter cultures. Restricted oxygen transfer may have further influenced the redox balance, plausibly reducing the efficiency of NAD^+^ regeneration required to sustain long-term lactic acid synthesis [13,16]. Similar viscosity-induced limitations of fermentation kinetics had been reported for systems enriched with oat β-glucan or curdlan, where the increased density of the matrix had diminished the metabolic activity of *Streptococcus thermophilus* and *Lactobacillus delbrueckii* ssp. *bulgaricus* [14]. The lower titratable acidity (≤41 °SH at 4 h) and the more gradual increase during storage (51.21–61.67 °SH after 28 days) had confirmed that SCGL had delayed both primary acidification and post-acidification by altering the physicochemical conditions that govern substrate accessibility. Increased viscosity and altered casein–polysaccharide interactions may have reduced lactose diffusivity and limited access of β-galactosidase to its substrate. These observations had corresponded with the documented effects of cereal β-glucans on fermented dairy systems. Qu et al. had shown that adding 0.3% oat β-glucan reorganised the protein network in a way that improved water retention, whereas increasing the level to 0.5% had weakened the gel and reduced its functional performance [11]. This pattern had mirrored the behaviour observed in our study: at 1% SCGL, the yoghurt matrix had become markedly more rigid, as reflected by elevated τ_0_ and k values, and this denser structure had likely limited mass transfer within the gel.

Lactic acid formation had been most pronounced during active fermentation, but its slower accumulation in SCGL-fortified yoghurts had indicated that the polysaccharide had moderated the glycolytic flux. SCGL also had affected the formation of secondary organic acids.

Acetic acid, produced via heterofermentative and redox-balancing pathways, had increased steadily in all samples; however, its concentrations had remained consistently lower in SCGL-containing variants. This pattern had suggested that SCGL may have reduced carbon diversion from pyruvate towards acetate-producing pathways, likely due to restricted metabolic branching within the more diffusion-limited gel structure and the moderated activity of *L. bulgaricus*. Similar reductions in acetate formation under polysaccharide-thickened conditions had been reported by Aljewicz et al. in yoghurts enriched with oat β-glucan and scleroglucan in low-fat yoghurts, where increased matrix density had led to measurable shifts in carbon flow and volatile-compound synthesis [13,14].

Citrate metabolism had followed a similar pattern. Citrate concentrations had decreased during fermentation, but this decline had been significantly attenuated in SCGL samples (*p* < 0.001; η^2^p > 0.94). Slower citrate utilisation had suggested that SCGL had affected citrate accessibility or had moderated the activity of citrate lyase. Since citrate catabolism contributes to the formation of key flavour compounds such as diacetyl and acetoin, its stabilisation in SCGL-enriched systems may have subtly influenced the yoghurt’s aromatic profile. Comparable effects of β-glucans, which induce phase separation and hinder metabolite exchange, had been described in previous studies [14].

These relationships had been supported by statistical analyses. Multivariate ANOVA (Wilks’ test) had confirmed significant effects of fermentation time, SCGL concentration and their interaction on acidification dynamics (*p* < 0.001; η^2^p > 0.89; test power = 1.0). One-way ANOVA further had shown significant effects of fermentation time and product type on lactic, acetic and citric acid concentrations (*p* < 0.001; η^2^p ≥ 0.96).

Overall, the results had demonstrated that SCGL had moderated the metabolic conversion of lactose into organic acids not by inhibiting LAB growth, but by altering the microstructural and diffusional properties of the yoghurt matrix. By increasing viscosity and limiting mass transfer, SCGL had slowed lactose hydrolysis, had reduced lactic and acetic acid formation, had attenuated citrate metabolism and had delayed post-acidification, while maintaining high viability of *S. thermophilus* and controlled activity of *L. bulgaricus*. These effects had not compromised fermentation completeness or product quality; instead, they had promoted a more stable and controlled fermentation system (Table 4).

### 2.7. Effect of Scleroglucan on the Content of Volatile Compounds

The aroma of yoghurt arose from a complex network of biochemical reactions during fermentation, including the metabolism of lactose, amino acids and lipids by starter cultures, which had generated numerous volatile and non-volatile compounds shaping the sensory profile of the final product [29]. Although more than one hundred volatiles had been identified in yoghurt [14], only a limited number such as acetaldehyde, 2,3-butanedione (diacetyl), acetoin, 2,3-pentanedione, 2-butanone and acetic acid had played a decisive role in defining its characteristic flavour. In full-fat yoghurt systems, milk fat further modulated aroma behaviour by influencing the solubility, partitioning and headspace release of hydrophobic volatiles.

**Figure 3:**
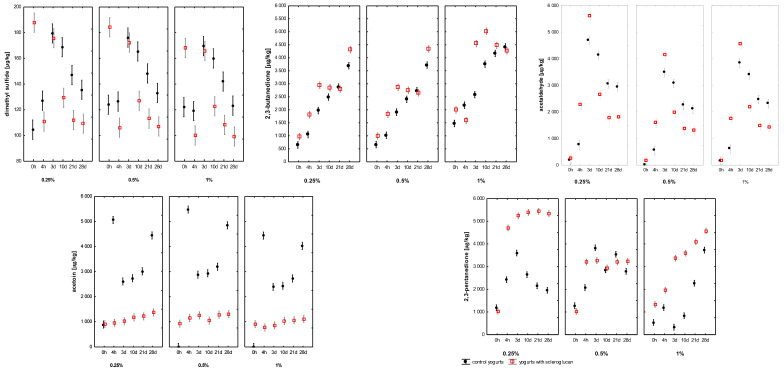
Changes in the volatile compound profiles of full-fat yoghurts containing skimmed milk powder (SMP; 0.25; 0.5; 1%, control samples, C) and scleroglucan (SCGL; 0.25; 0.5; 1%, experimental samples) during production and 28 days of refrigerated storage at 4 °C. Values are presented as means ± SD (*n* = 3). The 0–4 h period represents milk acidification, where 4 h marks the point when pH 4.6 was reached and refrigerated storage began.

The concentrations of the principal volatiles dimethyl sulphide (DMS), 2,3-butanedione, acetaldehyde, acetoin and 2,3-pentanedione are presented in Figure 3. DMS, responsible for subtle sulphurous notes, had ranged from 104 to 180 µg kg^−1^ in control samples and from 99 to 188 µg kg^−1^ in SCGL-containing samples. Its concentration had been significantly affected by storage time and SCGL concentration (*p* < 0.001), suggesting a modest influence of SCGL on sulphur-containing amino-acid metabolism, potentially reflecting altered redox or oxygen-transfer conditions within the modified gel structure. Notably, DMS levels in this study had been substantially lower and more stable than those reported for low-fat yoghurt enriched with SCGL [13], where the absence of fat and the use of skim milk powder had promoted higher precursor availability and stronger oxidative shifts, resulting in markedly elevated DMS accumulation.

2,3-butanedione (diacetyl), a key aroma-active diketone responsible for buttery notes, had increased throughout fermentation and storage in all samples, ranging from 647 to 4418 µg kg^−1^ in controls and from 971 to 5025 µg kg^−1^ in SCGL variants. SCGL concentration, storage time and their interaction had significantly influenced diacetyl accumulation (*p* < 0.001; η^2^p ≥ 0.71). Samples containing 0.25–0.5% SCGL had shown faster diacetyl increases, whereas 1% SCGL had produced a slower but more stable rise. These trends indicated that SCGL likely had modulated diacetyl diffusion and retention rather than its enzymatic synthesis. Unlike fermentable carbohydrate additives that had enhanced pyruvate metabolism [30], SCGL did not provide an additional carbon source; instead, its effects appeared consistent with structural modifications of the protein–polysaccharide matrix that had reduced molecular mobility. Compared with the low-fat system of Aljewicz et al. (2025), where diacetyl had shown a stronger dose-dependent shift and had reached higher end concentrations, the full-fat yoghurt matrix had moderated these changes, likely due to fat-partitioning effects that had buffered diketone accumulation [13].

In contrast, acetoin levels had been significantly lower in SCGL yoghurts (902–1374 µg kg^−1^) than in controls (up to 5063 µg kg^−1^), indicating that SCGL had reduced the enzymatic reduction of diacetyl to acetoin. This effect may have been associated with altered redox balance due to reduced oxygen availability or with diffusional constraints within the more viscous matrix. As a result, the elevated diacetyl-to-acetoin ratio in SCGL samples had contributed to a richer and more pronounced buttery note, consistent with sensory findings. A similar trend had been observed in SCGL-supplemented low-fat yoghurt [13], although the reduction in acetoin had been more pronounced in full-fat yoghurt, likely due to the additional influence of the lipid phase on oxygen solubility and redox potential.

Acetaldehyde, the key compound responsible for the yoghurt’s characteristic fresh, green note, ranged from 0 to 4697 µg kg^−1^ in control samples and 147 to 5587 µg kg^−1^ in those supplemented with SCGL. Significant effects of storage time, SCGL level and their interaction (*p* < 0.001; η^2^p ≥ 0.72), indicating that SCGL had influenced acetaldehyde behaviour primarily through retention rather than biosynthesis. Acetaldehyde is formed mainly from threonine, valine and pyruvate metabolism by *L. delbrueckii* subsp. *bulgaricus*, and there is no evidence that SCGL directly had stimulated these metabolic pathways. Instead, the polysaccharide’s hydrated microstructure may have reduced headspace migration of acetaldehyde through physical entrapment, a mechanism also described for other microbial EPS and β-glucans in fermented dairy matrices [14,30]. Relative to the low-fat yoghurt studied by Aljewicz et al. (2025), acetaldehyde levels in the present full-fat system had been lower and more stable, suggesting that the fat phase had acted as a barrier to volatile diffusion and had reduced the intensity of precursor-driven metabolic shifts observed in skim-milk matrices [13].

A similar effect had been observed for 2,3-pentanedione, whose concentrations had been consistently higher in SCGL-containing samples (1021–5439 µg kg^−1^) compared with controls (540–3831 µg kg^−1^). Given that diketones are prone to diffusion-limited losses from the gel matrix, their higher levels in SCGL yoghurts had further supported the interpretation that SCGL had enhanced volatile retention. However, in comparison with the low-fat yoghurt analysed by Aljewicz et al. (2025), where 2,3-pentanedione had accumulated more rapidly and had reached higher end values, the increases observed in full-fat yoghurt had been more moderate—again consistent with the dampening influence of the lipid phase on diketone mobility and headspace release [13].

When expressed in comparable units, diacetyl (≤5.0 mg L^−1^), acetaldehyde (≤5.6 mg L^−1^) and acetoin (≤5.0 mg L^−1^) concentrations had been within or slightly below the optimal sensory ranges reported by Tian et al. (2020) [31] for yoghurt matrices (6.65–9.12, 25.9–35.5, and 37.3–49.9 mg L^−1^, respectively). Importantly, that study had demonstrated that these compounds had exhibited synergistic enhancement when present in balanced ratios rather than high absolute quantities [31]. The volatile ratios observed in SCGL yoghurts—elevated diacetyl, moderate acetaldehyde and suppressed acetoin—had fitted this synergistic model, explaining the more rounded, creamy and fresh sensory perception.

Collectively, the incorporation of SCGL had significantly modulated both the formation and, more prominently, the retention of key volatile compounds. These effects appeared primarily governed by microstructural changes within the casein–fat–polysaccharide network rather than direct biochemical interactions. While specific SCGL–protein interactions were not measured in this study, the observed rheological and diffusional changes had aligned with mechanisms previously reported for β-glucans and microbial EPS forming hydrated, three-dimensional networks that had reinforced the gel structure and had limited mass transfer [13,30]. Such networks had reduced volatile diffusion to the headspace by increasing viscosity and decreasing molecular mobility. Therefore, the stabilising effects of SCGL on volatile profiles likely had stemmed from its ability to modify the physical matrix rather than from specific binding or chemical interactions with aroma molecules. This interpretation was consistent with the behaviour observed in both full-fat and low-fat yoghurt systems, while also explaining the attenuated magnitude of volatile

### 2.8. Effect of Scleroglucan Addition on the Sensory Properties of Yoghurt

The mean values of the sensory attributes of the control yoghurts and yoghurts enriched with SCGL are presented in Table 5. Sensory attributes were divided into five groups associated with appearance, aroma, consistency, mouthfeel, and taste. The overall acceptability of the produced yoghurts was also assessed.

In the group of appearance-related attributes, the first attribute evaluated was colour uniformity, which was found to be very uniform across all yoghurts, regardless of the proportions of SMP or SCGL added (*p* > 0.05). Yoghurts enhanced with SCGL were characterised by a slightly creamy colour and were slightly less creamy than the controls (*p* < 0.05). Whey separation was most pronounced in the control yoghurts. However, no whey separation was observed in yoghurts containing SCGL. Thereby, the sensory analysis confirmed the results relating to syneresis presented in Table 2. Also, the available literature confirms that various types of β-glucans can be used as effective water-holding agents in food products, primarily categorised by their source and structure. The most effective types are oat, barley, and yeast β-glucans, as well as the fungal exopolysaccharide scleroglucan [32].

The yoghurt aroma was most intense in the control sample containing 0.25% SMP. In the other control yoghurts, the aroma was slightly less intense but still distinct. In the experimental yoghurts, however, the intensity of the yoghurt aroma decreased with a rise in SCGL concentration (*p* < 0.05). This difference was clearly discernible, that the yoghurt aroma intensity was rated as weak in the 1% SCGL variant.

A difference in the sour aroma was also observed (*p* < 0.05). The control yoghurts formed a homogeneous group and were rated as having a strongly sour smell. The yoghurt with 0.25% SCGL addition was described as moderately sour. In contrast, the sour aroma was rated as faint in the 0.5% and 1% SCGL variants. None of the assessed yoghurts had a sweet or foreign aroma.

All yoghurts differed in uniformity, lumpiness, and thickness of consistency (*p* < 0.05). The 0.25% SCGL variant had the least uniform consistency, being the lumpiest and thinnest of all samples. The control yoghurt containing 0.25% SMP had a very uniform consistency with moderate lumpiness, whereas the other samples exhibited similarly uniform consistency but negligible or no lumpiness. All control yoghurts were described as thick. The average consistency ratings increased slightly with a rise in SMP addition. The thickness of the experimental yoghurts also increased with increasing SCGL concentrations, but the observed changes were more pronounced. As mentioned earlier, the 0.25% SCGL variant was thin; the 0.50% SGL variant was moderately thick, whereas the yoghurt with the highest concentration of SCGL was described as very thick. However, this study reveals that scleroglucan, in comparison, for example, to pullan, has a stronger thickening effect at a 1% addition, while pullan is a strong thickener at a 2% addition [33]. Moreover, the thickening effect of scleroglucan depends on the fat content in the raw material. In yoghurts produced from skimmed milk, the thickening effect of scleroglucan at concentrations of 0.25% and 0.50% is stronger than in full-fat yoghurts [13].

In the next step, the sensory panel evaluated the attributes related to mouthfeel. Statistically significant differences were observed in adhesiveness, smoothness, and mealiness (*p* < 0.05). The control yoghurts were generally characterised by moderate adhesion to the palate, strong or very strong smoothness, and very low mealiness. In turn, the adhesiveness and smoothness of the experimental yoghurts were described as weak in the 0.25% SCGL variant, moderate in the 0.50% SCGL variant, and very strong in the 1% SCGL variant. In contrast, the perceived mealiness of the experimental yoghurts decreased with increasing SCGL concentrations.

The assessment of taste attributes revealed significantly higher intensity of yoghurt and sour tastes in the controls than in the samples with SCGL addition. The typical yoghurt taste was described as moderately detectable in the 0.25% SCGL variant and as only faintly detectable in the other two. The sour taste was moderately noticeable in yoghurts with 0.25% and 0.50% addition of SCGL and was weakly perceptible in the yoghurt containing 1% SCGL. Similar results for yoghurt with added β-glucan were obtained by other authors. Beta-glucan addition at 0.75 and 0.9% levels caused a statistically considerable decrease in yoghurt and acid flavour [34]. The sweet taste was weakly perceptible in all products but slightly stronger in the 1% SCGL and 1% SMP variants. No bitter or foreign tastes were detected in any of the tested products.

The overall acceptability of yoghurts was assessed in the final stage of the sensory analysis. The control yoghurts, characterised by a more intense yoghurt flavour and aroma than those with SCGL addition, were highly rated. Among the experimental yoghurts, the 1% SCGL variant received the highest acceptability rating, although the panellists described it as more of a dessert than a yoghurt owing to its thick consistency, the absence of whey separation, and a faint yoghurt taste. In turn, yoghurts containing 0.25% and 0.50% SCGL received low ratings, mainly due to their rather thin consistency and high mealiness. Unlike other β-glucans [35], SCGL added to whole milk-yoghurts at lower concentrations did not act as a thickening agent, but it completely eliminated whey separation.

## 3. Materials and Methods

### 3.1. Materials

Raw cow’s milk was sourced from a local dairy plant in Olsztyn, Poland. Fermentation was carried out using a YC-180 commercial yoghurt starter culture (Chr. Hansen, Hørsholm, Denmark) containing *Streptococcus thermophilus* and *Lactobacillus delbrueckii* subsp. *bulgaricus*. The milk composition was standardised with SMP supplied by Mlekpol (Mrągowo, Poland). The experimental yoghurts were fortified with highly purified SCGL (90% purity; Mw = 0.97 × 10^6^ g/mol) isolated from *Sclerotium rolfsii* (Cargill, Krefeld, Germany). The scleroglucan preparation used in this study is a microbial β-(1→3)(1→6)-D-glucan obtained from *Sclerotium rolfsii*, characterised by a β-(1→3)-linked backbone with regularly occurring β-(1→6)-linked single-glucose side chains (branching ratio approx. 3:1), as documented in earlier physicochemical characterisation studies [9]. The reference compounds, including [^2^H_6_]dimethyl sulphide, [^13^C_4_]2,3-butanedione, [^2^H_4_]acetaldehyde, d-glucose, d-galactose, lactose monohydrate, hexane, and H_2_SO_4_, were obtained from Sigma-Aldrich (St. Louis, MO, USA).

### 3.2. Experimental Design

Two separate datasets were collected during this study. The first dataset described the yoghurt fermentation process, where “0 h” denoted the milk immediately after inoculation with the starter culture (before fermentation), and “4 h” represented the end of fermentation, when the product reached pH 4.6 and the yoghurt gel had formed. The second dataset referred to refrigerated storage. After fermentation, the yoghurt was transferred to refrigerated conditions, initiating the storage period. Samples were collected after 3, 10, 21, and 28 days of storage.

### 3.3. Yoghurt Production

Yoghurt was prepared under controlled laboratory conditions following the method outlined in patent PL235801. Milk was acidified using the YC-180 starter culture (Chr. Hansen, Hørsholm, Poland). Whole raw cow’s milk (4.21% fat, 3.87% protein; pH 6.78) was obtained from a local dairy plant in Olsztyn, Poland. In the production hall of the Dairy Processing and Quality Management Department (University of Warmia and Mazury in Olsztyn), the milk was heated to 45 °C and separated using a cream separator (Spomasz LWG-20, Gniezno, Poland) to obtain skimmed milk. The skimmed phase was then continuously standardised to a fat content of 3.2% by blending it with the appropriate amount of cream. The standardised milk contained 3.2% fat and 3.94% protein.

A specified amount of SCGL (0.25–1% *w*/*w*) or an equivalent amount of SMP (0.25–1% *w*/*w*; control) was then added. The mixture was stirred for 30 min at 45 °C, heated to 60 °C, and maintained under continuous agitation for 1 h. The milk was subsequently homogenised at 140/10 bar (Panda, GEA, Parma, Italy), pasteurised at 90 °C for 10 min, cooled to 43 °C using a plate heat exchanger (Alfa Laval P20-HB, Lund, Sweden), and inoculated with the YC-180 starter culture at 0.02 g per 100 g of milk.

The inoculated mixture was transferred into sterile 100 mL containers, sealed, and incubated at 43 °C until the pH reached 4.6 (approximately 4 h). After fermentation, the yoghurts were cooled to 4 °C and stored at this temperature for further analyses. The final products were packaged in 100 mL cups (Ø57 × H76 mm), with separate containers prepared for each analytical determination.

### 3.4. Microbiological Analysis

Microbiological enumeration was conducted according to EN ISO 7218:2024 [36]. *Streptococcus thermophilus* counts were determined on M17 agar (Merck, Darmstadt, Germany) after aerobic incubation at 45 °C for 48 h, whereas *Lactobacillus delbrueckii* ssp. bulgaricus were enumerated on Rogosa agar (Merck, Darmstadt, Germany) after anaerobic incubation at 37 °C for 72 h using the AnaeroGen system (Oxoid, Poznan, Poland).

### 3.5. Acidification Monitoring

Acidification kinetics was monitored at 1 min intervals and a temperature of 43 °C using a microelectrode connected to the Cerko Lab System (Cerko Lab, Gdynia, Poland). The measurements were continued until the pH of the sample reached 4.6.

### 3.6. Isolation of Volatile Compounds

Volatile compounds were analysed according to a procedure described by Aljewicz et al. (2020), with some modifications [14]. For each analysis, 14 g of yoghurt was placed in a 20 mL glass vial, spiked with isotopically labelled internal standards ([^2^H_6_]dimethyl sulphide, [^13^C_4_]2,3-butanedione, and [^2^H_4_]acetaldehyde) to a final concentration of 300 ppb, and sealed with a PTFE/silicone septum. Volatiles were extracted from the headspace by solid-phase microextraction (SPME) using a carboxen/polydimethylsiloxane (CAR/PDMS) fibre (Supelco, St. Louis, MO, USA). Automatic extraction was performed using a CTC CombiPAL autosampler (Agilent Technologies, Santa Clara, CA, USA). The samples were equilibrated at 50 °C for 5 min, after which the SPME fibre was exposed to the headspace for 45 min and subsequently desorbed in the GC injector port at 260 °C for 5 min. Injections were performed in splitless mode with a 1 min purge. Gas chromatographic separation and detection were performed using a comprehensive two-dimensional gas chromatograph coupled with time-of-flight mass spectrometry (GC × GC-ToF-MS; Pegasus IV, LECO, St. Joseph, MI, USA). The system was equipped with an SPB-5 capillary column (30 m × 0.32 mm × 0.25 µm) as the primary column and a Supelcowax 10 column (1 m × 0.10 mm × 0.10 µm) as the secondary column. The carrier gas was helium, flowing at 0.8 mL s^−1^. The injector temperature was set to 220 °C, and the secondary oven and modulator were maintained at offsets of +5 °C and +15 °C, respectively, relative to the primary oven. The temperature programme started at 40 °C (held for 2 min), increased to 230 °C at 5 °C min^−1^, and held for 5 min. The modulator period was 3 s, and the MS detector operated in electron ionisation mode (70 eV) with a mass scan range of *m*/*z* 33–330, acquisition rate of 150 scans s^−1^, and detector voltage of 1750 V. Compounds were identified by matching mass spectra and calculating retention indices (RI) against the NIST 2017 library and authentic reference standards. Retention indices were determined using a homologous series of n-alkanes (C_7_–C_20_) in hexane. Quantification was performed based on the peak-area ratios of analytes to their corresponding labelled internal standards (Table 6) with ChromaTOF software ver. 4.4 (LECO). The results were expressed in µg kg^−1^ of yoghurt. 

Because the labelled internal standards are physicochemically identical to their native analytes and fully compensate for extraction efficiency, matrix effects, and ionisation variability, SIDA does not require conventional multi-point external calibration. In practice, a single-point calibration (based on the fixed concentration of the isotopically labelled internal standard) is sufficient for accurate quantitation.

We also emphasise that SIDA is widely recognised as the most accurate and precise method for the quantification of volatile organic compounds, as it intrinsically corrects for sample preparation losses and instrumental fluctuations, ensuring robust and reliable results.

### 3.7. Lactic Acid and Citric Acid Determination

Citric acids were quantified by HPLC according to Aljewicz et al. (2025) [13]. Lactic acid was extracted according to a procedure proposed by Ferreira Barros et al. [37], with some modifications. Briefly, 1 mL of the sample was combined with 5 mL of 0.05 M H_2_SO_4_, vortexed, and incubated at 40 °C for 1 h. The samples were centrifuged at 5000× *g* for 30 min at 4 °C, filtered through 0.45 µm RC syringe filters, and analysed by HPLC (Agilent 1260) with a DAD detector (215 nm) using a Supelcosil 610H cation-exchange column (300 × 7.8 mm) (Sigma-Aldrich, Bellefonte, PA, USA) and a H^+^ microguard cartridge at 60 °C. The mobile phase was 0.05 M H_2_SO_4_, delivered isocratically at 0.5 mL min^−1^. Quantification was performed using external standards and quantified by external calibration (R^2^ = 0.9991) for lactic acid and (R^2^ > 0.99) for citric acid. Note: In milk, citric acid is predominantly present as citrate anions. 

### 3.8. Lactose Analysis

Lactose was analysed according to Aljewicz et al. (2020) [14]. Briefly, one millilitre of the sample was combined with 5 mL of Milli-Q^®^ water (Millipore, Burlington, MA, USA), vortexed, incubated at 40 °C for 1 h, centrifuged at 5000× *g* for 30 min at 4 °C, filtered, and injected (20 µL, in triplicate) into the HPLC system (Agilent 1260) with ELSD detection (gain 9, evaporation/nebulisation 50 °C, 3.5 bar). Separation was achieved using a Supelcosil 610H column with a H^+^ microguard at 30 °C. The mobile phase was Milli-Q^®^ water, delivered at 1 mL min^−1^. Quantification was performed using external standards and quantified by external calibration (R^2^ = 0.9991).

### 3.9. Sensory Analysis

The sensory analysis of the control yoghurts (containing SMP) and the experimental yoghurts (containing SCGL) was conducted in the University’s sensory laboratory meeting the requirements of the EN ISO 8589:2010 standard [38], using the profiling method described in EN ISO 13299:2016-05E [39]. The sensory panel consisted of eight experts (scientists) with at least 10 years of experience in dairy product sensory evaluations who were selected, trained, monitored, and possessed appropriate sensory sensitivity in accordance with EN ISO 8586:2012 [40]. Before the main sensory test, three 2 h sessions were conducted for the panellists with the use of commercial yoghurts, milk, cream, milk powder, and flavour substances. Yoghurt samples with the addition of SMP and SCGL were coded using a three-digit random number and served in 100 mL transparent hinged-led propylene containers at 20 °C. The yoghurt samples were served randomly, but in the same order for all assessors. The test was conducted in a single session lasting approximately 1 h, with a 1–2 min intervals to rinse the mouth with water between samples. The definition card included 18 sensory attributes which intensity was assessed on a five-point scale, where 1 denoted the absence of a given attribute, and 5 denoted very strong intensity. The overall acceptability of the tested yoghurts was also assessed. The test was conducted 10 days after production. Polish legislation does not require the approval from a bioethics committee in case of sensory evaluation studies involving food products.

### 3.10. Statistical Analysis

The results were verified for normal distribution and homogeneity of variance. The data were presented as means ± standard deviation. The significance of differences between means was analysed using Tukey’s test, and the interactions between factors (storage time, concentration of additives, and presence of β-glucans) were assessed by analysis of variance (ANOVA). The results of the sensory analysis were processed by one-way ANOVA and Fisher’s LSD test. The experiment was conducted in duplicate. Statistical analyses were conducted using Statistica 13.5 PL software (Statsoft 2017, Krakow, Poland) at a significance level of α = 0.05 (*n* = 3).

## 4. Conclusions

This study demonstrated that scleroglucan (SCGL) effectively modulates the behaviour of full-fat yoghurt systems by influencing fermentation dynamics, supporting starter culture activity, and improving the physicochemical and sensory characteristics of the final product. Its ability to reinforce the protein matrix, stabilise the fat–water interface, and eliminate syneresis confirms its role as a natural, multifunctional structuring agent. The comprehensive analytical approach applied here provides new insights into how SCGL interacts with the milk matrix. Future research should include extended storage trials and an evaluation of SCGL in yoghurt systems containing additional starter cultures, particularly probiotic strains, to determine whether this β-glucan can simultaneously enhance structural stability and support microbial viability. From an industrial standpoint, SCGL represents a promising alternative to starch or carrageenan, offering clean-label stabilisation while contributing potential health benefits associated with β-glucans.

## Figures and Tables

**Table 1:** Chemical composition full-fat yoghurts containing skimmed milk powder (SMP; 0%, 0.25%, and 1%; control samples, C) and scleroglucan (SCGL; 0.25%, 0.5%, and 1%; experimental samples) after production.

Sample	Concentration [%]	Fat Content (%) ± SD	Dry Matter Content (DM, %) ± SD	Protein Content (%) ± SD
Control	0.25	3.2 ± 0.02	14.32 ^b^ ± 0.11	4.23 ^a^ ± 0.02
SCGL		3.19 ± 0.02	14.75 ^a^ ± 0.11	3.82 ^c^ ± 0.01
Control	0.5	3.21 ± 0.02	14.11 ^c^ ± 0.02	4.08 ^b^ ± 0.02
SCGL		3.20 ± 0.01	14.45 ^ab^ ± 0.04	3.76 ^c^ ± 0.04
Control	1	3.18 ± 0.02	13.94 ^d^ ± 0.05	3.64 ^d^ ± 0.01
SCGL		3.20 ± 0.01	13.95 ^d^ ± 0.06	3.57 ^d^ ± 0.03

Values are presented as means ± SD (*n* = 3). Different superscript letters in columns indicate significant differences at *p* ≤ 0.05.

**Table 2:** The functional properties of control and experimental full-fat yoghurts during 28 days of refrigerated storage. The values 0.25%, 0.5%, and 1% indicate the concentrations of SCGL in the yoghurt samples.

Product	Control				SCGL			
Time [Days]	3	10	21	28	3	10	21	28
0.25%								
Syneresis [%]	2.34 ^c^ ± 0.24	4.47 ^b^ ± 0.15	6.08 ^a^ ± 0.53	5.05 ^b^ ± 0.38	0 ± 0	0 ± 0	0 ± 0	0 ± 0
Hardness [N]	1.77 ± 0.23	1.88 ± 0.1	2.08 ± 0.15	2.04 ± 0.09	3.6 ± 0.01	3.31 ± 0.41	3.84 ± 0.46	3.89 ± 0.16
Adhesiveness [N·s]	−13.86 ± 5.87	−12.38 ± 1.48	−13.66 ± 2.99	−11.59 ± 0.97	−6.89 ^b^ ± 1.12	−11.52 ^a^ ± 0.99	−15.35 ^a^ ± 4.16	−15.96 ^a^ ± 4.66
L	87.81 ^b^ ± 0.12	88.15 ^a^ ± 0.04	85.67 ^c^ ± 0.16	85.62 ^c^ ± 0.19	76.67 ^c^ ± 0.18	85.24 ^a^ ± 0.1	83.72 ^b^ ± 0.2	83.66 ^b^ ± 0.12
a	−1.87 ^b^ ± 0.03	−1.93 ^c^ ± 0.02	−1.78 ^a^ ± 0.03	−1.91 ^ab^ ± 0.01	−2.63 ^c^ ± 0.01	−1.91 ^b^ ± 0.04	−1.86 ^a^ ± 0.02	−1.95 ^b^ ± 0.02
b	7.23 ^c^ ± 0.07	7.6 ^a^ ± 0.07	7.34 ^bc^ ± 0.04	7.47 ^ab^ ± 0.14	6.83 ^c^ ± 0.08	7.21 ^b^ ± 0.1	7.02 ^c^ ± 0.03	7.31 ^b^ ± 0.04
0.50%								
Syneresis [%]	3.94 ^b^ ± 0.49	4.3 ^b^ ± 0.61	4.83 ^b^ ± 0.73	6.81 ^a^ ± 0.22	0 ± 0	0 ± 0	0 ± 0	0 ± 0
Hardness [N]	2.21 ^c^ ± 0.09	2.36 ^b^ ± 0.07	2.43 ^ab^ ± 0.03	2.52 ^a^ ± 0.07	5.15 ^b^ ± 0.21	5.58 ^a^ ± 0.16	5.66 ^a^ ± 0.18	5.57 ^a^ ± 0.18
Adhesiveness [N·s]	−11.93 ± 1.19	−11.73 ± 0.29	−9.9 ± 1.15	−10.94 ± 1.52	−11.33 ± 0.42	−11.6 ± 1.09	−11.96 ± 3.27	−11.89 ± 0.3
L	86.78 ^ab^ ± 0.18	86.95 ^a^ ± 0.18	86.5 b ± 0.13	85.89 ^c^ ± 0.07	82.21 ^b^ ± 0.22	82.76 ^a^ ± 0.09	82.3 ^b^ ± 0.14	82.31 ^b^ ± 0.1
a	−1.95 ^b^ ± 0.02	−1.9 a ± 0.01	−1.98 b ± 0.03	−1.97 ^b^ ± 0.02	−2.11 ^a^ ± 0.02	−2.15 ^b^ ± 0.01	−2.17 ^b^ ± 0.01	−2.1 ^a^ ± 0.02
b	7.61 ^a^ ± 0.04	7.46 b ± 0.05	7.58 a ± 0.03	7.58 ^a^ ± 0.05	7.39 ^b^ ± 0.04	7.44 ^b^ ± 0.04	7.6 ^a^ ± 0.02	7.44 ^b^ ± 0.07
1.00%								
Syneresis [%]	7.49 ^a^ ± 0.28	7.01 ^a^ ± 0.66	7.23 ^a^ ± 0.35	5.23 ^b^ ± 0.41	0 ± 0	0 ± 0	0 ± 0	0 ± 0
Hardness [N]	2.36 ^c^ ± 0.08	2.71 ^b^ ± 0.09	2.8 ^ab^ ± 0.03	2.89 ^a^ ± 0.09	5.17 ^b^ ± 0.3	6.74 ^a^ ± 0.09	6.86 ^a^ ± 0.05	6.84 ^a^ ± 0.04
Adhesiveness [N·s]	−9.7 ^ab^ ± 0.16	−8.13 ^a^ ± 0.72	−8.09 ^a^ ± 0.35	−12.28 ^b^ ± 3.73	−16 ^b^ ± 1.6	−17.64 ^b^ ± 0.22	−19.18 ^a^ ± 1.62	−19.22 ^b^ ± 2.89
L	87.01 ^a^ ± 0.05	86.17 ^b^ ± 0.45	85.22 ^c^ ± 0.22	85.29 ^c^ ± 0.11	85.4 ^a^ ± 0.34	75.94 ^b^ ± 0.09	75.98 ^b^ ± 0.11	76.14 ^b^ ± 0.06
a	−1.7 ^ab^ ± 0.01	−1.69 ^a^ ± 0.04	−1.75 ^b^ ± 0.02	−1.71 ^ab^ ± 0.02	−1.82 ^a^ ± 0.04	−2.74 ^b^ ± 0.02	−2.76 ^b^ ± 0.02	−2.86 ^c^ ± 0.01
b	7.33 ± 0.03	7.33 ± 0.05	7.37 ± 0.04	7.33 ± 0.04	7.28 ^a^ ± 0.17	6.94 ^b^ ± 0.2	7.43 ^a^ ± 0.05	7.57 ^a^ ± 0.15

Values are presented as means ± SD (*n* = 3). Different superscript letters in rows denote significant differences at *p* ≤ 0.05.

**Table 3:** Rheological properties of full-fat yoghurts containing skimmed milk powder (SMP; 0%, 0.25%, and 1%; control samples, C) and scleroglucan (SCGL; 0.25%, 0.5%, and 1%; experimental samples) during 28 days of refrigerated storage. HB—Herschel–Bulkley model; τ_0_—yield stress; η—apparent viscosity at shear rates of 10, 50, and 100 s^−1^; flow index (*n*) and consistency coefficient (k).

	0.25%				0.5%				1%			
	C 3	C 28	SCGL 3	SCGL 28	C 3	C 28	SCGL 3	SCGL 28	C 3	C 28	SCGL 3	SCGL 28
HB τ_0_	0.000	0.000	221.251	340.409	0.000	0.000	304.391	468.326	0.000	0.000	480.755	739.674
HB k	3688.4	3167.8	1266.7	2828.100	3231.400	2853.200	3247.200	2474.154	2417.300	2469.300	1463.000	923.430
HB *n*	0.01	0.01	0.01	0.01	0.010	0.010	0.010	0.010	0.010	0.010	0.010	0.010
R^2^ HB	0.995	0.988	0.979	0.989	0.986	0.853	0.899	415.200	0.925	0.985	0.985	0.989
η at 10 SR [1/S]	46.900	48.300	128.000	88.800	37.700	50.600	53.600	87.900	38.000	32.500	13.000	14.100
η at 50 SR [1/S]	12.500	12.500	30.100	20.100	10.100	12.100	13.600	20.300	10.300	8.600	3.650	3.600
η at 9 SR [1/S]	6.290	6.440	15.000	9.970	5.350	5.950	6.480	10.500	5.270	4.480	2.010	1.880

Values are presented as means ± SD (*n* = 3).

**Table 4:** The organic acid and lactose content of full-fat control and experimental yoghurts during production and storage. The values 0.25%, 0.5%, and 1% indicate the concentration of scleroglucan (SCGL) in the experimental yoghurt samples.

Product	Control					SCGL				
Time	Production [Hours]		Storage [Days]			Production [Hours]		Storage [Days]		
	0	4	3	10	28	0	4	3	10	28
0.25%										
Acetic acid (mg·kg^−1^)	404.96 ^ab^ ± 2.54	6617.6 ^f^ ± 267.8	9651.4 ^g^ ± 184.7	11,066 ^h^ ± 178.4	12,312 ^i^ ± 655.0	0.0 ^a^ ± 0.0	704.8 ^b^ ± 34.9	1735.1 ^c^ ± 130.9	3410.9 ^d^ ± 205.2	4144.9 ^e^ ± 275.6
Citrate (g·kg^−1^)	0.379 ^c^ ± 0.003	0.365 ^a^ ± 0.005	0.363 ^ab^ ± 0.000	0.360 ^ab^ ± 0.003	0.362 ^ab^ ± 0.002	0.365 ^a^ ± 0.007	0.365 ^a^ ± 0.001	0.360 ^a^ ± 0.004	0.362 ^ab^ ± 0.006	0.358 ^ab^ ± 0.002
Lactic acid (g·kg^−1^)	0.0471 ^a^ ± 0.0004	0.329 ^e^ ± 0.002	0.367 ^b^ ± 0.002	0.399 ^g^ ± 0.000	0.428 ^h^ ± 0.007	0.047 ^a^ ± 0.001	0.225 ^d^ ± 0.003	0.342 ^f^ ± 0.001	0.368 ^b^ ± 0.001	0.375 ^c^ ± 0.001
Lactose (g·kg^−1^)	50.13 ^c^ ± 0.91	47.71 ^b^ ± 0.74	46.82 ^a^ ± 0.20	46.39 ^a^ ± 0.57	46.59 ^a^ ± 0.44	49.57 ^bc^ ± 0.43	48.44 ^b^ ± 0.75	46.81 ^a^ ± 0.38	46.92 ^a^ ± 0.57	46.52 ^a^ ± 0.07
0.5%										
Acetic acid (mg·kg^−1^)	51.25 ^a^ ± 1.16	4306.3 ^e^ ± 319.8	8669.3 ^d^ ± 131.4	8648.6 ^d^ ± 337.7	10,459 ^e^ ± 378.7	0.0 ^a^ ± 0.0	633.5 ^a^ ± 41.2	1522.8 ^b^ ± 169.5	3045.5 ^c^ ± 90.2	3634 ^ce^ ± 222.1
Citrate (g·kg^−1^)	0.400 ^c^ ± 0.002	0.379 ^a^ ± 0.001	0.388 ^a^ ± 0.001	0.377 ^a^ ± 0.003	0.378 ^a^ ± 0.002	0.388 ^b^ ± 0.004	0.377 ^a^ ± 0.002	0.375 ^a^ ± 0.002	0.377 ^a^ ± 0.001	0.378 ^a^ ± 0.002
Lactic acid (g·kg^−1^)	0.049 ^b^ ± 0.001	0.246 ^e^± 0.005	0.373 ^a^ ± 0.001	0.392 ^f^ ± 0.001	0.413 ^g^ ± 0.004	0.049 ^b^ ± 0.001	0.214 ^d^ ± 0.009	0.351 ^c^ ± 0.001	0.359 ^c^ ± 0.001	0.379 ^a^ ± 0.001
Lactose (g·kg^−1^)	49.90 ^c^ ± 0.90	48.26 ^b^ ± 0.74	46.69 ^a^ ± 0.20	46.38 ^a^ ± 0.57	46.63 ^a^ ± 0.44	49.24 ^bc^ ± 0.43	48.23 ^b^ ± 0.75	46.44 ^a^ ± 0.38	46.70 ^a^ ± 0.56	46.21 ^a^ ± 0.07
1%										
Acetic acid (mg·kg^−1^)	0.0 ^a^ ± 0.0	1736.3 ^c^ ± 53.8	2179.6 ^d^ ± 197.1	2335.9 ^d^ ± 149.9	5177.9 ^f^ ± 292.3	0.0 ^a^ ± 0.0	1004.6 ^b^ ± 9.6	1056.9 ^b^ ± 55.2	1126.6 ^b^ ± 64.4	3098.7 ^e^ ± 57.4
Citrate (g·kg^−1^)	0.401 ^b^ ± 0.004	0.399 ^b^ ± 0.009	0.395 ^b^ ± 0.002	0.391 ^ab^ ± 0.006	0.401 ^b^ ± 0.003	0.395 ^b^ ± 0.002	0.384 ^a^ ± 0.002	0.384 ^a^ ± 0.003	0.383 ^a^ ± 0.002	0.385 ^a^ ± 0.002
Lactic acid (g·kg^−1^)	0.053 ^a^ ± 0.001	0.230 ^b^ ± 0.009	0.349 ^d^ ± 0.001	0.370 ^e^ ± 0.002	0.390 ^g^ ± 0.001	0.053 ^a^ ± 0.000	0.226 ^b^ ± 0.003	0.338 ^c^ ± 0.001	0.363 ^e^ ± 0.000	0.384 ^f^ ± 0.001
Lactose (g·kg^−1^)	49.88 ^d^ ± 0.90	48.42 ^c^ ± 0.75	46.90 ^ab^ ± 0.20	46.57 ^ab^ ± 0.58	46.83 ^ab^ ± 0.45	48.60 ^cd^ ± 0.42	47.54 ^bc^ ± 0.74	45.98 ^a^ ± 0.37	46.10 ^a^ ± 0.56	45.61 ^a^ ± 0.07

Values are presented as means ± SD (*n* = 3). Different superscript letters in rows denote significant differences at *p* < 0.05.

**Table 5:** The mean values of the sensory attributes of control (C) and experimental (S) full-fat yoghurt samples containing scleroglucan. The results are presented as means (*n* = 8).

Sensory Attributes	C 0.25%	C 0.5%	C 1%	SCGL 0.25%	SCGL 0.5%	SCGL 1%	*p*-Value
**Appearance**							
Colour uniformity	4.6	4.6	4.6	4.7	4.7	4.8	>0.05
Creamy colour	3.2 ^a^	3.2 ^a^	3.3 ^a^	2.9 ^b^	2.9 ^b^	2.6 ^b^	0.000
Whey separation	3.7 ^a^	3.5 ^a^	3.5 ^a^	1.1 ^b^	1.0 ^b^	1.0 ^b^	0.000
**Aroma**							
Typical of yoghurt	4.5 ^a^	3.7 ^b^	3.7 ^b^	2.8 ^c^	2.2 ^d^	1.5 ^e^	0.000
Sour	3.4 ^a^	3.4 ^a^	3.2 ^a^	2.1 ^b^	1.7 ^c^	1.5 ^c^	0.000
Sweet	1.0	1.0	1.0	1.0	1.0	1.0	>0.05
Foreign	1.0	1.0	1.0	1.0	1.0	1.0	>0.05
**Consistency**							
Uniform	3.9 ^b^	4.3 ^a^	4.1 ^a^	1.4 ^c^	4.1 ^ab^	4.0 ^ab^	0.000
Lumpy	2.4 ^b^	1.0 ^d^	1.4 ^c^	4.1 ^a^	1.0 ^d^	1.6 ^c^	0.000
Thick	3.3 ^b^	3.8 ^a^	3.9 ^a^	1.8 ^d^	2.6 ^c^	4.1 ^a^	0.000
**Mouthfeel**							
Adhesiveness	2.5 ^b^	2.4 ^b^	2.7 ^b^	1.4 ^c^	2.6 ^b^	4.1 ^a^	0.000
Smoothness	3.3 ^b^	4.2 ^a^	4.1 ^a^	1.3 ^d^	2.5 ^c^	4.2 ^a^	0.000
Mealiness	1.4 ^c^	1.1 ^c^	1.1 ^c^	4.1 ^a^	3.0 ^b^	1.3 ^c^	0.000
**Taste**							
Typical of yoghurt	4.0 ^a^	3.9 ^a^	3.6 ^a^	2.7 ^b^	1.9 ^c^	1.5 ^d^	0.000
Sour	3.6 ^a^	3.6 ^a^	3.4 ^a^	2.6 ^b^	2.5 ^b^	1.9 ^c^	0.000
Sweet	1.1 ^b^	1.2 ^b^	1.8 ^a^	1.2 ^b^	1.3 ^b^	1.9 ^a^	0.001
Bitter	1.0	1.0	1.0	1.0	1.0	1.0	>0.05
Foreign	1.0	1.0	1.0	1.0	1.0	1.0	>0.05
**Overall** **Acceptability**	4.0 ^ab^	4.2 ^ab^	4.3 ^a^	1.5 ^d^	2.1 ^c^	3.9 ^b^	0.005

^a,b,c,d,e^—Mean values in rows marked with different letters differ significantly at *p* ≤ 0.05.

**Table 6:** Labelled standards and quantification ions used in the stable isotope dilution assay (SIDA).

Compound	Quant. Ions (*m*/*z*) ^a^	Labelled Standard	Ion IS (*m*/*z*) ^b^
Dimethyl sulphide	62	^2^H_6_-Dimethyl sulphide	68
2,3-Butanedione	86	^13^C_4_-2,3-Butanedione	90
Acetaldehyde	44	^2^H_4_-Acetaldehyde	48
2,3-Pentanedione	100	^13^C_4_-2,3-Butanedione	90

^a^ Ions of analytes used for quantification. ^b^ Ions of internal standards (labelled isotopes) used for quantification.

## Data Availability

The original contributions presented in this study are included in the article. Further inquiries can be directed to the corresponding author.
